# Evidence on Measures for the Prevention of Pressure Injuries in Mechanically Ventilated Patients in Prone Positioning: A Systematic Review

**DOI:** 10.3390/healthcare14040443

**Published:** 2026-02-10

**Authors:** Simone Amato, Daniele Napolitano, Alessio Lo Cascio, Elena Conoscenti, Angela Lappa, Emilio D’avino, Mirko Masciullo, Antonello Pucci, Valentina De Bartolo, Claudia Torretta, Lucia Mitello, Anna Rita Marucci, Francesco Gravante

**Affiliations:** 1 Cardiac Intensive Care Unit, Heart Transplant Centre and ECMO Service, San Camillo Forlanini Hospital, 00152 Rome, Italy; mirkomasciullo@gmail.com; 2 Centre for Digestive Diseases (CEMAD), Fondazione Policlinico Universitario A. Gemelli IRCCS, 00168 Rome, Italy; daniele.napolitano@policlinicogemelli.it; 3 Direction of Health Professions, La Maddalena Cancer Center, 90146 Palermo, Italy; 4 Department of Internal Medicine and Pediatrics, Ghent University, 9000 Ghent, Belgium; elena.conoscenti@ugent.be; 5 IRCCS ISMETT, 90127 Palermo, Italy; 6 UPMC Italy, 90133 Palermo, Italy; 7 Department of Heart and Vessels, Cardiovascular Intensive Care Unit, San Camillo Forlanini Hospital, 00152 Rome, Italy; anglap.3112@gmail.com (A.L.); edavino@scamilloforlanini.rm.it (E.D.); 8 Shock and Trauma Unit, San Camillo Forlanini Hospital, 00152 Rome, Italy; antonello.pucci@uniroma1.it; 9 Multispecialty Surgery Unit, European Hospital, 00149 Rome, Italy; valesingha@gmail.com; 10 Department of Health Professions, San Camillo Forlanini Hospital, 00152 Rome, Italy; ctorretta@scamilloforlanini.rm.it (C.T.); lmitello@scamilloforlanini.rm.it (L.M.); ar.marucci@gmail.com (A.R.M.); 11 Intensive Care Unit, Department of Critical Care, Local Health Authority of Caserta, 81100 Caserta, Italy; fra.gravante83@gmail.com

**Keywords:** prone positioning, pressure injuries, critical care, intensive care nursing, prevention strategies, prophylactic dressings, skin integrity, care bundles

## Abstract

Background: Therapeutic prone positioning is widely used to improve oxygenation in patients with acute respiratory distress syndrome but is associated with an increased risk of pressure injuries, particularly affecting facial and anterior body regions. Methods: This systematic review was conducted according to PRISMA 2020 and Joanna Briggs Institute guidelines and was prospectively registered in PROSPERO (CRD42023442604). PubMed, CINAHL, Web of Science, Scopus, and the Cochrane Library were searched from inception to June 2025, including grey literature. Primary studies involving adult, mechanically ventilated patients undergoing therapeutic prone positioning and evaluating pressure injury prevention strategies were included. Methodological quality was assessed using JBI critical appraisal tools. Owing to clinical and methodological heterogeneity, findings were synthesized using a Synthesis Without Meta-analysis (SWiM) approach. Results: Eight studies with heterogeneous designs were included. Preventive interventions mainly comprised prophylactic dressings, repositioning and support devices, and comprehensive care bundles. Most strategies were associated with a reduction in pressure injury incidence, particularly in facial and anterior anatomical areas. Greater effectiveness was observed when interventions were implemented within structured protocols supported by staff training and multidisciplinary coordination. Conclusions: Preventive strategies appear effective in reducing pressure injuries associated with prone positioning in critically ill patients. The implementation of standardized, bundled prevention protocols may improve patient safety in intensive care settings.

## 1. Introduction

Pressure injuries, also referred to as decubitus ulcers, ischemic ulcers, bedsores, or pressure injuries (PIs), are injuries to the skin and underlying tissues that commonly occur over bony prominences or in areas exposed to sustained pressure from medical devices [1,2]. According to the National Pressure Injuries Advisory Panel (NPIAP), individuals at high risk of PI development often present multiple contributing factors that influence both the mechanical conditions surrounding tissue loading and their intrinsic susceptibility and tolerance [3,4]. Among the most recognized risk factors are low or high body weight or body mass index (BMI), the use of vasoactive and inotropic drugs, and cardiovascular comorbidities such as heart failure, all of which can impair tissue perfusion and increase vulnerability to skin breakdown [5,6,7].

These factors converge and are amplified in critically ill patients, whose pathophysiological state is characterized by hemodynamic instability, prolonged sedation, immobility, mechanical ventilation, and reliance on invasive medical devices [8,9]. In this milieu, the complex interaction between systemic compromise and sustained external pressure predisposes these patients to an elevated risk of PI formation, posing distinct challenges for effective prevention and clinical management [10].

ICU-specific pressure injury prevention requires tailored strategies, including frequent repositioning, pressure-redistributing surfaces, vigilant monitoring of skin and device interfaces, nutritional optimization, and early mobilization as tolerated.

Although many hospital-acquired PIs are preventable through evidence-based practices such as risk stratification and consistent implementation of these interventions [11,12], achieving adherence in the ICU remains challenging due to the complexity of critical illness and high-intensity therapeutic demands. Furthermore, traditional risk assessment tools are often subjective and not specifically designed for critically ill patients; as a result, they may inadequately capture true pressure injury risk, highlighting the need for more objective, ICU-specific assessment methods [11].

Effective prevention in this setting therefore requires a multifaceted approach, including frequent repositioning, specialized support surfaces, vigilant skin and device site monitoring, early mobilization when feasible, and nutritional optimization with input from dietitians [10].

Despite advancements in healthcare delivery, PIs remain a major global public health concern and a critical threat to patient safety [12]. In particular, hospital-acquired PIs in the ICU are frequently categorized as never events due to their preventability and are associated with considerable morbidity [13,14].

The global burden is considerable: PIs affect millions of patients worldwide, with an estimated prevalence ranging from 10% to 20% among those admitted to the ICU [15,16]. This prevalence may reach 30% in patients undergoing therapeutic prone positioning (PP) due to increased and prolonged pressure on vulnerable tissues [17]. The risk and burden of PIs are markedly higher in ICU patients compared to those in general wards [18]. Critically ill patients are more likely to experience device-related PIs in less typical anatomical locations such as the face, chest, or limbs due to prolonged immobility and technological dependency [10]. The global DecubICUs study, a multicenter point prevalence study across 90 countries, found that 26.6% of ICU patients had at least one PI, with 16.2% developing them during their ICU stay [19]. These figures highlight the ongoing need for targeted interventions in this vulnerable population.

Among the ICU-specific interventions contributing to the development of PIs is therapeutic PP. This technique is widely used to improve oxygenation and reduce mortality in patients with moderate-to-severe acute respiratory distress syndrome (ARDS), as demonstrated in the landmark PROSEVA trial [20,21]. By promoting more even ventilation–perfusion distribution and reducing lung injury, PP is a critical component of ARDS management [22,23]. However, maintaining patients in the PP for prolonged periods of ≥16 consecutive hours, as recommended by the ESICM guidelines, substantially increases the risk of PIs, particularly in anterior regions such as the face, thorax, and knees, which are in constant contact with support surfaces [17,24].

This increased risk is not only due to external mechanical loading, but also to impaired microcirculation, tissue deformation, and reduced cellular tolerance to ischemia in critically ill patients [25]. While several observational and experimental studies have identified an association between PP and PIs, results remain heterogeneous, and few have systematically examined effective preventive strategies specific to PP patients [26]. A systematic review and meta-analysis confirmed a significantly higher risk of PIs in PP compared to those in supine positions [27]. Yet, the evidence base remains limited, with a pressing need for high-quality research on tailored prevention strategies in this context [20].

### Systematic Review Objective

This systematic review aims to evaluate the effectiveness of available strategies for preventing and reducing the risk of PIs in critically ill patients undergoing therapeutic PP.

## 2. Methods

### 2.1. Design and Reporting Standards

This systematic review was conducted in accordance with the Preferred Reporting Items for Systematic Reviews and Meta-Analyses (PRISMA) guidelines [28] and the Joanna Briggs Institute (JBI) Manual for Evidence Synthesis [29]. The protocol was prospectively registered in the International Prospective Register of Systematic Reviews (PROSPERO) [30,31] (Registration ID: CRD42023442604).

### 2.2. Research Question and Eligibility Criteria

The research question was formulated using a modified PIO framework:

- Population (P): Critically ill adult patients with ARDS undergoing therapeutic PP while intubated and receiving invasive mechanical ventilation;
- Intervention (I): Strategies for the prevention of PI;
- Outcome (O): Skin integrity and incidence of PI including mucous membrane PI.

Studies were included if they met the following criteria: primary research studies involving adult patients (≥18 years) receiving therapeutic PP, focused on interventions or strategies to prevent PI or maintain skin integrity; publications in English, Italian, Spanish, Portuguese, or French were included without date restriction.

Specifically, we included intubated, mechanically ventilated adults with ARDS undergoing therapeutic PP in ICU settings.

Studies were excluded if they focused exclusively on pediatric populations or did not address strategies to prevent PIs in patients undergoing PP.

We also excluded studies on awake (non-intubated) PP and studies evaluating PP in surgical/operating-room contexts.

Additionally, case series, case reports, editorials, opinion pieces, study protocols, conference abstracts, and scoping or systematic reviews were not considered eligible.

Studies describing PP protocols or feasibility without evaluating preventive interventions or pressure injury outcomes were excluded.

Articles published in languages for which reliable translation was not feasible were also excluded from the analysis.

### 2.3. Search Strategy

A methodological approach based on a comprehensive search across the following databases was adopted: PubMed, CINAHL, Web of Science, Scopus, and the Cochrane Library, covering all the literature up to June 2025. Supplementary manual searches were conducted in Google Scholar and through reference lists of relevant articles [32].

Search strategies combined MeSH terms and free-text keywords, using Boolean operators (AND, OR). Terms included “acute respiratory distress syndrome”, “prone position”, “pressure ulcer”, “skin integrity”, “pressure injury”, and their synonyms. Full search strategies are available in Table S1.

### 2.4. Study Selection

All records were imported into Rayyan^®^ (Rayyan Systems Inc., Cambridge, MA, USA) [33] for deduplication and blinded screening. Two reviewers (AP and MM) independently assessed titles, abstracts, and full texts. Discrepancies were resolved through discussion or adjudicated by a third reviewer (SA).

### 2.5. Data Extraction

A structured data extraction form was developed to systematically collect the following variables from each included study: author(s), year, and country; study design; sample size and patient characteristics; clinical outcomes measured; medications used and medication management strategies; protocols and procedures applied; timing and duration of PP; main findings; and direction of effect. Pressure injury assessments were conducted as reported in the original studies; in all cases, evaluations were performed by trained healthcare professionals according to the criteria or classification systems adopted by each study and at the time points specified within the individual study protocols.

For clarity, a positive effect direction was defined as an outcome that was statistically significant according to the *p*-value reported by the study authors, while a negative effect direction was defined as an outcome that was not statistically significant.

### 2.6. Methodological Quality Appraisal

The methodological quality of the included studies was assessed using the appropriate JBI Critical Appraisal Tools, selected according to each study’s design [34]. Two reviewers (AP and MM) independently evaluated all studies, and any discrepancies were resolved through discussion or, if necessary, by consulting a third reviewer (SA). Based on the JBI scores, studies were classified as high-quality when scoring above 70%, moderate-quality when scoring between 50 and 69.9%, and low-quality when scoring below 50%. Results are presented in Table S1.

### 2.7. Level of Evidence

Each study was also appraised using the Oxford Centre for Evidence-Based Medicine (OCEBM) 2011 Levels of Evidence (OCEBM, 2011), classifying evidence from Level 1 (e.g., systematic reviews of RCTs) to Level 5 (e.g., expert opinion).

### 2.8. Data Synthesis

Given the clinical and methodological heterogeneity of included studies, a meta-analysis was not feasible. Instead, a Synthesis Without Meta-analysis (SWiM) approach was applied [35]. This decision is consistent with previous observational evidence highlighting substantial variability in pressure injury incidence, anatomical distribution, and preventive approaches among prone-positioned intensive care patients [36].

This approach was deemed methodologically appropriate given the heterogeneity in study designs, interventions, and outcome measures. Studies were grouped by intervention type (e.g., prophylactic dressings, repositioning protocols, skin care regimens). Outcomes were narratively summarized by effect direction (positive or not significant), with attention to frequency, context, and clinical relevance. This synthesis strategy ensured transparent integration of findings across diverse study designs and settings.

## 3. Results

### 3.1. Selection Process

**Figure 1:**
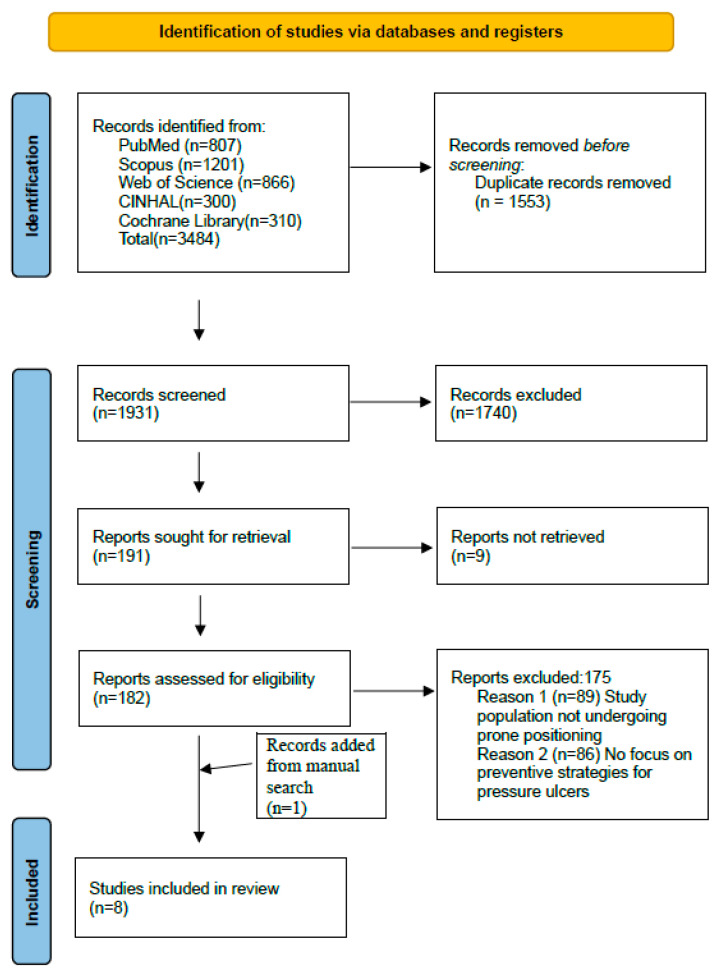
PRISMA flow diagram of the review process.

A total of 3484 records were identified through database searches. After removing duplicates and screening titles and abstracts, 191 reports were sought for retrieval. Nine reports were not retrieved due to the unavailability of the full text despite attempts to access them through institutional subscriptions and direct search; and 182 full-text articles were assessed for eligibility. Of these, 175 were excluded for not focusing on prone positioning or pressure injury prevention strategies. One additional study was retrieved manually, resulting in eight studies being included in the final synthesis (see Figure 1).

### 3.2. Study Characteristics

The eight included studies were published between 2005 and 2025 and were conducted in various countries, including Australia, France, China, Germany, Austria, the United States, and Ireland. The study designs included one randomized controlled trial [37], two quasi-experimental studies [38,39], three cohort studies [40,41,42], one prospective observational study [43], and one retrospective case–control study [44]. Sample sizes ranged from 8 to 156 critically ill, mechanically ventilated adult patients undergoing therapeutic PP for ARDS. All studies evaluated the effectiveness of preventive strategies for reducing the risk of PIs, with particular attention to high-risk anatomical areas such as the face, chin, chest, and forehead. Reported interventions included prophylactic dressings, specialized support surfaces, repositioning protocols, and structured care bundles. Further methodological details, intervention specifics, and study outcomes are summarized in Table 1.

### 3.3. Quality of Included Studies and Level of Evidence

The methodological quality of the included studies ranged from 73% to 89%, with no studies excluded based on critical appraisal results. One randomized controlled trial demonstrated high quality: Gay et al. [37] scored 77%. The quasi-experimental studies also reported strong methodological rigor: Ge et al. [40] scored 89%, McEvoy et al. [39] and Prebio et al. [38] scored 78%, while Woolger et al. [43] and Liu et al. [44] each scored 78% and 89%, respectively.

Among the cohort studies, Otto et al. [41] and Stéphan et al. [42] both received 73%.

Details of the critical appraisal are presented in Table 2. According to the OCEBM scoring system, four studies were classified as level 3 evidence, three studies as level 2 evidence, and only one study as level 1 evidence (Table 3).

### 3.4. Intervention Strategies

The included studies evaluated a range of interventions aimed at preventing PIs in critically ill patients undergoing therapeutic PP. These strategies were categorized into three main types: prophylactic dressings, repositioning and support devices, and comprehensive care bundles.

### 3.5. Prophylactic Dressings

Several studies assessed the effectiveness of prophylactic dressings in protecting vulnerable anatomical areas such as the face, chin, forehead, and chest. Otto et al. [41] applied multilayer silicone foam dressings (Mepilex^®^ Border Flex, Mölnlycke Health Care, Gothenburg, Sweden), reporting a significant reduction in PI incidence on the chin and forehead among 49 patients. Ge et al. [40] observed a lower PI incidence when soft silicone foam dressings were used across repeated prone sessions.

### 3.6. Repositioning and Support Devices

Mechanical strategies were implemented to offload pressure during PP. Prebio et al. [38] tested the Prone Head Support (PHS) system, reducing the severity of perioral ulcers. Liu and Tang [44] combined two-hour head repositioning with pressure-relieving pads and dressings, leading to fewer PIs and extubation-related complications. Stéphan et al. [42] integrated eye lubrication (carbomer gel), eyelid taping, and reverse Trendelenburg positioning with scheduled repositioning, effectively preventing ocular injuries and skin breakdown.

### 3.7. Comprehensive Care Bundles

Comprehensive bundles showed strong preventive outcomes. McEvoy et al. [39] implemented a protocol including dressings, frequent skin inspections, head mobilization, and alternating support devices, reducing PI incidence. Gay et al. [37], through a randomized trial, confirmed the efficacy of structured bundles integrating early head rotation, foams, and staff training. Woolger et al. [43] emphasized protocol adherence, noting better outcomes when compliance exceeded 80%.

## 4. Clinical Outcomes by Intervention Type

### 4.1. Facial Area (Cheeks, Chin, Forehead)

Facial PIs were commonly reported at bony prominences. Otto et al. [41] reported a significantly lower incidence of facial PIs in patients treated with silicone adhesive multilayer foam dressings, with a statistically significant reduction at the chin (44.0% vs. 16.7%, *p* = 0.038) compared with patients not receiving prophylactic foam protection.

### 4.2. Ocular Region

Stéphan et al. [42] showed that combining eye lubrication, taping, and repositioning every four hours prevented keratopathy in proned COVID-19 patients.

### 4.3. Oral and Perioral Areas

Prebio et al. [38] showed lip ulcer prevention using PHS for pronation > 6 h.

### 4.4. Anterior Chest and Bony Prominences

While most studies focused on facial and perioral injuries, some also addressed anterior thoracic regions and pressure-prone bony prominences. McEvoy et al. [39] included dressings and offloading strategies for the chest and iliac crests within a structured bundle, reporting reduced PI incidence. Liu and Tang [44] applied pressure-relieving pads over the chest and pelvis, noting decreased skin breakdown. Otto et al. [41] described the use of silicone dressings on anterior sites, though without outcome stratification. These findings highlight the importance of protecting not only the face but also the anterior torso and skeletal prominences during prolonged PP.

### 4.5. Generalized Outcomes

Liu and Tang [44] reported a reduction in PIs and device-related complications using combined repositioning and dressings. McEvoy et al. [39] and Gay et al. [37] demonstrated that multidisciplinary bundles lowered PI incidence and improved patient outcomes.

### 4.6. Management and Application of Anti-Decubitus Interventions

Effective PI prevention requires integrating interventions into routine workflows. Prebio et al. [38] demonstrated the feasibility of the Prone Head Support (PHS) system in ICU patients undergoing PP. Otto et al. [41] highlighted the role of nurse training in dressing application. Liu and Tang [44] reinforced regular repositioning and skin monitoring. McEvoy et al. [39] stressed that multidisciplinary coordination enhances compliance and feasibility.

**Figure 2:**
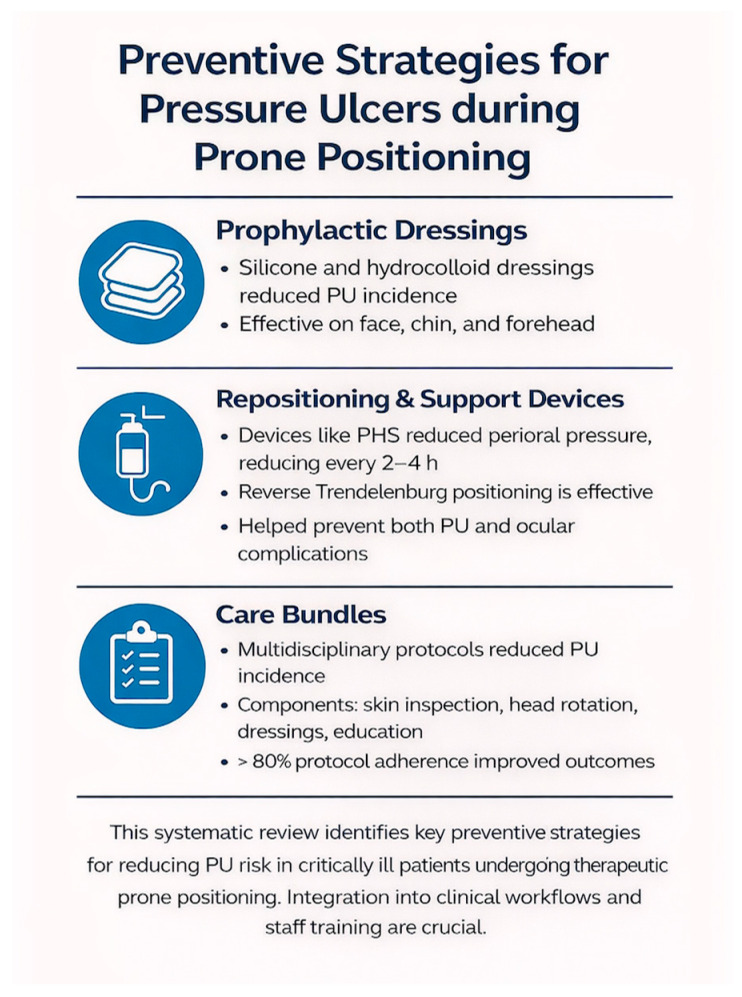
Preventive Strategies for Pressure Ulcers in Critically Ill Patients during Prone Positioning. Note. This infographic summarizes the main interventions dressings, support devices, and care bundles effective in preventing PUs in critically ill patients during prone positioning.

The key findings are summarized and visually represented in Figure 2.

## 5. Discussion

This study aimed to identify strategies described in the literature for preventing and reducing the risk of PI in critically ill, mechanically ventilated patients undergoing therapeutic PP, with a focus on evaluating the effectiveness of these strategies. Analysis of the included studies revealed three main categories of interventions commonly adopted for PI prevention: (1) prophylactic dressings, (2) repositioning and support devices, and (3) comprehensive care bundles. These interventions should be interpreted with regard to their clinical impact, mechanisms of action, and contextual feasibility, rather than solely as statistically significant outcomes.

Beyond the general benefits and challenges of PP, our review focuses on pragmatic prevention strategies, such as dressings, support devices, positioning techniques, and structured bundles, applicable to intubated, mechanically ventilated ARDS patients. Prophylactic dressings and structured care bundles appear most effective, likely because they simultaneously address multiple risk factors such as pressure redistribution, moisture control, and monitoring by trained staff. Repositioning and support devices showed variable effectiveness, possibly due to differences in implementation or study design.

These observations indicate that combining multiple strategies within bundled approaches may optimize PI prevention in high-risk ICU patients.

Conflicting evidence exists regarding the optimal duration of PP [45,46]. While duration is not a direct outcome for PI prevention in our review, consideration of PP timing is clinically relevant for safely implementing preventive strategies [47,48,49]. Careful attention to patient tolerance and risk profile remains essential. Several subgroup analyses in meta-analyses [50,51] demonstrated a survival benefit with pronation lasting at least 12 h, whereas other studies [27,52] proposed different cutoffs ranging from 10 to 16 h. In line with the recent ESICM guidelines, durations of ≥16 consecutive hours are recommended to achieve optimal outcomes [24].

Recent ICU practice has been influenced by next-generation preventive materials and devices specifically tailored for prolonged proning in intubated ARDS patients. Silicone multilayer foam dressings applied systematically to high-risk facial sites (chin, forehead) were associated with a marked reduction in facial injuries in observational and pre/post studies [41]. In parallel, positioning and support solutions such as the Prone Head Support system and pressure redistribution air mattresses reduced lesion extent and severity in early randomized and pilot work [38], while refinements technique like the ‘Swimmer’s Position’ further lowered overall PI incidence compared with traditional ‘Face-Down’ positioning [44].

These findings underline that intervention effectiveness is context-dependently influenced by adherence to correct technique, staff training, and device selection.

Bundled approaches that combine these elements with structured skin inspection and staff training showed improvements in severe-injury profiles, though randomized evidence remains mixed [37,38,39]. Collectively, these data highlight the necessity of integrating device selection, prophylactic dressings, positioning techniques, and staff training in standardized PP prevention pathways.

Guérin et al. [21] further supported the notion that each additional hour in the PP may contribute to improved outcomes, although durations exceeding 16 h require further investigation. Implementing structured, multifaced strategies is essential to reduce complications in long-term ICU survivors.

PIs have a significant negative impact on patient outcomes, particularly affecting psychological well-being [47] and quality of life after discharge [48]. PP redistributes pressure to areas not commonly exposed to sustained load, increasing the risk of PIs, as confirmed by meta-analyses and reviews [27,49]. The use of dedicated PI prevention devices should therefore be considered a foundational component of comprehensive care rather than optional. In this context, the use of dedicated PI prevention devices should be considered a foundational approach [50].

It is also important to consider the pathophysiological and clinical complexity of patients requiring PP. These individuals often present severe hypoxemia associated with hemodynamic instability and require advanced support measures such as vasopressors, deep sedation, and mechanical ventilation. Recent evidence has shown that the use of vasopressor agents is significantly associated with an increased risk of PIs due to compromised tissue perfusion and capillary ischemia [9]. Moreover, extremes in BMI, including both underweight and obese profiles, have been identified as independent risk factors for PI development during hospitalization, with a nonlinear dose–response relationship that underscores the need for personalized preventive strategies [51].

These findings reinforce that the effectiveness of interventions may vary depending on patient severity and comorbidities, highlighting the importance of tailoring prevention strategies. Early identification and targeted prevention in high-risk ICU population are therefore critical.

Among the most effective strategies identified, the implementation of standardized care bundles comprising evidence-based practices delivered collectively proved particularly beneficial in the context of PP, especially when including skin protection measures, scheduled repositioning, and targeted staff education, thereby contributing to a significant reduction in PI incidence and enhancement of patient safety.

Lane et al. [53], through a systematic review and meta-analysis, compared alternating-pressure and low-air-loss support surfaces in ICU patients and concluded that although neither modality was significantly superior, both remain essential components within PI prevention bundles. This underscores the importance of integrating support surfaces into broader preventive strategies rather than viewing them in isolation.

The physiological impact of PP also warrants consideration. Hering et al. [54] observed an increase in intra-abdominal pressure during pronation. Weig et al. [55] highlighted the association between obesity and higher rates of multiorgan dysfunction during PP, particularly hepatic and renal impairments, although mortality rates were unaffected. Conversely, De Jong et al. [56] found that oxygenation improved more in obese patients than in non-obese ones.

To optimize early detection and intervention, Uslu et al. [57] evaluated the COMHON Index, an ICU-specific pressure injury risk assessment tool, and demonstrated its high interrater reliability among ICU nurses. The study supports the use of targeted screening tools to complement clinical judgment, especially in complex PP cases where standard tools like the Braden scale may be insufficient. These findings highlight that intervention effectiveness is enhanced when risk assessment is accurate and tailored to patient-specific factors.

The literature also emphasizes that PP should be performed in highly specialized environments with adequately trained personnel [29]. However, a cross-sectional survey by Song et al. [58] among ICU nurses in China revealed that low prioritization of PI prevention, lack of training, and systemic barriers contributed to suboptimal adherence to prevention guidelines. Leadership support and structured education emerged as key facilitators of improved clinical practice, emphasizing that staff education directly influences preventive strategy effectiveness.

Standardized protocols and coordinated multidisciplinary approaches are essential components to ensure safety and reduce complications. Overall, the review highlights that while individual interventions have variable success, their effectiveness is maximized when integrated into bundled, context-sensitive, and staff-supported care pathways.

Future research should prioritize large, multicenter randomized trials focused on the competencies of ICU professionals. Identifying training gaps and implementing targeted education could enhance intervention effectiveness and mitigate long-term complications in ICU survivors. A broader perspective may include the integration of technological, organizational, and procedural innovations aimed at optimizing pressure injury prevention during prolonged PP.

## 6. Limitations

This systematic review presents several limitations. First, the heterogeneity of study designs, intervention types, and outcome measurements limited the possibility of conducting a meta-analysis. Moreover, our findings indicate that the majority of the studies provide level 3 evidence, which suggests that the generalization of the results should be approached with caution. Although Embase was not included due to limited institutional access, this limitation was partially mitigated through comprehensive grey literature searches and manual reference screening, which are recognized strategies to enhance retrieval completeness in systematic reviews. In addition, several clinically relevant factors related to prone positioning, such as patient age, bladder care, and positioning strategies to prevent nerve injuries (e.g., brachial plexus injury), could not be systematically evaluated because they were inconsistently reported or not addressed in the included studies. Nonetheless, the review has notable strengths, including the systematic selection of peer-reviewed articles, the application of a standardized methodological quality assessment, and the inclusion of a reasonable number of patients, enhancing the robustness of the narrative synthesis.

## 7. Implications for Clinical Practice

The findings of this review support the integration of standardized strategies into routine ICU care pathways to reduce the adverse outcomes associated with PP. Healthcare institutions should implement protocols that include preventive measures for PIs, staff education, and monitoring tools tailored to high-risk patients. Specialized centers should also invest in structured training programs to enhance the competencies of ICU healthcare workers, ensuring the safe and effective delivery of prone therapy and improving patient outcomes after ICU discharge.

## 8. Conclusions

This review identified a range of strategies for prevention and reduction in PI risk in critically ill, mechanically ventilated patients undergoing therapeutic PP in ICU.

The main intervention types included prophylactic dressings, repositioning and support devices, and comprehensive care bundles. While PP remains a well-established therapy for patients with ARDS, it is associated with minor but clinically relevant complications, including facial edema and a heightened incidence of PIs.

The systematic implementation of preventive measures along with structured training for healthcare professionals emerges as a key factor in minimizing long-term adverse outcomes. Overall, the findings highlight the importance of integrating prophylactic dressings, structured repositioning strategies, and standardized care bundles into routine ICU practice to mitigate pressure injury risk during PP.

Future research should prioritize large-scale, randomized multicenter trials to validate these strategies and to identify educational gaps in ICU nursing and multidisciplinary staff.

## Figures and Tables

**Table 1:** Main characteristics of the included studies.

Author, Year, Country	Study Design	Patients (N Sample)	Outcome	Medications Used	Medication Management	Protocol/Procedures	Timing Prone Positioning	Main Finding	Results (+, NS)
Liu H et al., (2020) China [44]	Retrospective case–control	130. Observation group 65 vs. control group 65	- Hypotension, unplanned extubation -PI - Facial edema - Catheter removal - Corneal injury	Preventive plates (NS)	Head elevated 15–30°; horseshoe-shaped head ring used to prevent facial edema and eye injury	Intervention group (*n* = 65): repositioning every 2 h; head/limb adjustments; pressure point monitoring; airway and ventilator management	NS	Significantly fewer facial PI in the intervention group (*p* < 0.05)	+
Stéphan S et al., (2021) France [42]	Cohort study	20. (40 eyes)	-Ocular surface lesions (keratitis, chemosis)	Carbomer eye gel	The eyes were kept closed, and the nursing team administered an ophthalmologic lubricant (carbomer eye gel) twice daily	In intubated, mechanically ventilated adults with ARDS undergoing PP, eyelids were taped and lubricated; weekly ocular surface assessments were performed	Median duration of PP of 32 h Median PP 32 h (IQR 18–45)	Low incidence of corneal ulcers; no significant worsening from PP	+
McEvoy N et al., (2022) Ireland [11]	Quasi-experimental study	40	Incidence, severity and location of PI	Closed gel ring, barrier cream, and redistribution air mattress	Head rotations, use of a closed gel ring to promote endotracheal tube stability, use of barrier cream, and use of an air mattress for redistribution	Frequent head turns, gel head ring, prophylactic dressings, air mattress, and staff training for early PI detection	Pre-intervention for a total of 53.3 ± 21.6 h. For the post-intervention cohort 83.6 ± 45.4 h	PI incidence reduced by 25% post-intervention; fewer severe ulcers (no grade III/IV or deep tissue injuries post	+
Prebio M et al., (2005) Austria [38]	Quasi-experimental study	8	Pressure lesions on the lips	PHS (Prone Head Support system) with soft medical foam (Corpoform^®^)	Foam-padded facial mask with unobstructed pressure-free zones; height-adjustable suspension system at bed head	180° intubated, mechanically ventilated adults with ARDS undergoing PP with facial mask support; head fixed, arms repositioned every 4 h; mirror ensured airway visibility	>6 h a day	PHS system significantly reduced size and severity of pressure sores, especially on face and neck	+
Otto P et al., (2022) Germany [41]	Cohort study	49	PI on the chin and forehead	Silicone adhesive multilayer foams, (Mepilex^®^ border flex)	Foam dressings applied to chin and forehead before pronation in 25 patients; 25 received no dressings	Use of foam dressings to prevent PI	~123 h (mean ≈7375 min total prone time)	Significant reduction in facial PI after foam use; especially at chin (44% vs. 16.7%, *p* = 0.038)	+
Ge G et al., (2025) China [40]	Quasi-experimental	65	Incidence and site of pressure injuries during PP	Eye film (for eyelid protection)	Single-use application to closed eyes; details NR	2 h repositioning, head/shoulder elevation, horseshoe ring, eye care, suctioning, and skin protection	NS (standard ARDS duration presumed ≥ 12 h)	Postural nursing significantly reduced facial PI compared to routine care	+
Gay L et al., (2025) France [37]	RCT	156	Incidence and severity of PI in prone intubated, mechanically ventilated adults with ARDS undergoing PP	Methylcellulose eye gel; foam pads	Applied before PP; replaced every 24 h	Eye gel and eyelid taping, 15-degree body tilt, foam pads applied to chin and forehead, head repositioned every 4 h, and skin inspection every 12 h	PP for ≥16 h/day	PI incidence lower in intervention group, but not statistically significant (OR 0.92, CI 0.39–2.18)	NS
Woolger C et al., (2024) Australia [43]	Prospective observational study	63	Incidence and severity of PI across different prone techniques	NS	Facial support devices selected per patient anatomy; no dressings reported	Compared “Face Down” vs. “Swimmer’s Position”; face support devices selected per patient anatomy	Median 16 h/day	“Swimmer’s Position” significantly reduced incidence and severity of facial pressure injuries vs. “Face Down” position	+

Abbreviations: NS: not specific; PP: prone position; PI: pressure injuries; RCT: randomized controlled trial; NPUAP: National Pressure Ulcer Advisory Panel classification; ARDS: acute respiratory distress syndrome. In Results: “+” indicates a beneficial effect in PI prevention; “NS” denotes non-significant results; and IQR is interquartile range.

**Table 2:** Assessment of the quality of the included studies.

Study Analysed with The Joanna Briggs Institute Critical Appraisal Checklist for Cohort Studies															
Author and Year	Study Design	Q1	Q2	Q3	Q4	Q5	Q6	Q7	Q8	Q9	Q10	Q11			JBI Score (%)
Stéphan S. et al. (2021) [42]	Cohort study	+	+	+	+	U	+	U	+	+	U	+			8/11 (73)
Otto P et al. (2022) [41]	Cohort study	+	+	+	U	U	U	+	+	+	+	+			8/11 (73)
Woolger C. et al. (2024) [43]	Prospective observational study	+	+	+	N	U	+	+	+	+	+	U			8/11 (73)
Study analysed with The Joanna Briggs Institute critical appraisal checklist for quasi-experimental study															
Author and years	Study design	Q1	Q2	Q3	Q4	Q5	Q6	Q7	Q8	Q9					JBI Score (%)
McEvoy N. et al. (2022) [11]	Quasi-experimental study	+	+	+	U	+	+	+	+	U					7/9 (78)
Prebio M et al. (2005) [38]	Quasi-experimental study	+	+	+	U	+	+	+	+	U					7/9 (78)
Ge G. et al. (2025) [40]	Quasi-experimental study	+	+	+	N	+	+	+	+	+					8/9 (89)
Liu H et al. (2020) [44]	Retrospective case–control	+	+	+	+	U	+	+	+	+					8/9 (89)
Study analysed with The Joanna Briggs Institute critical appraisal checklist for randomized controlled trials															
Author and year	Study design	Q1	Q2	Q3	Q4	Q5	Q6	Q7	Q8	Q9	Q10	Q11	Q12	Q13	JBI Score (%)
Gay L et al. (2025) [37]	RCT	+	+	+	U	U	U	+	+	+	+	+	+	+	10/13 (77)

Note: + = yes, - = no, U = unclear, N = not applicable, JBI = Joanna Briggs Institute; RCT = randomized controlled trial.

**Table 3:** Level of evidence.

Author, Year, Country	Study Design	OCEBM Level
Liu H et al. (2020), China [44]	Retrospective case–control study	3
Stéphan S et al. (2021), France [42]	Prospective cohort study	2
McEvoy N et al. (2022), Ireland [11]	Quasi-experimental study	3
Prebio M et al. (2005), Austria [38]	Quasi-experimental study	3
Otto P et al. (2022), Germany [41]	Cohort study	3
Ge G et al. (2025), China [40]	Quasi-experimental	3
Gay L et al. (2025), France [37]	RCT	1
Woolger C et al. (2024), Australia [43]	Prospective observational study	3

## Data Availability

No new data were created or analyzed in this study.

## Supplementary Materials

The following supporting information can be downloaded at: https://www.mdpi.com/article/10.3390/healthcare14040443/s1, Table S1: Search strategy.
