# Supplementary material for: Evidence on Measures for the Prevention of Pressure Injuries in Mechanically Ventilated Patients in Prone Positioning: A Systematic Review
Source: Healthcare (Basel). 2026 Feb 10;14(4):443. doi: 10.3390/healthcare14040443 (PMC12941124; doi:10.3390/healthcare14040443)
Supplement: Supplementary file 1 [file healthcare-14-00443-s001.zip › healthcare-4111450-supplementary.pdf]

Supplementary file 1

Table S1. Search strategy

Pubmed: n 807

#1	("distress syndrome respiratory"[Title/Abstract] OR "distress syndromes respiratory"[Title/Abstract] OR "respiratory distress syndromes"[Title/Abstract] OR "syndrome respiratory distress"[Title/Abstract] OR "Shock Lung"[Title/Abstract] OR "lung shock"[Title/Abstract] OR "respiratory distress syndrome acute"[Title/Abstract] OR "Acute Respiratory Distress Syndrome"[Title/Abstract] OR "ARDS"[Title/Abstract])
#2	("Prone Positions"[Title/Abstract] OR "Prone Position"[Title/Abstract] OR "pron*"[Title/Abstract])
#3	("Pressure Ulcer"[Title/Abstract] OR "Pressure Ulcers"[Title/Abstract] OR "Ulcer"[Title/Abstract] OR "Pressure"[Title/Abstract] OR "bedsore*"[Title/Abstract] OR "decubit*"[Title/Abstract])
#4	("distress syndrome respiratory"[Title/Abstract] OR "distress syndromes respiratory"[Title/Abstract] OR "respiratory distress syndromes"[Title/Abstract] OR "syndrome respiratory distress"[Title/Abstract] OR "Shock Lung"[Title/Abstract] OR "lung shock"[Title/Abstract] OR "respiratory distress syndrome acute"[Title/Abstract] OR "Acute Respiratory Distress Syndrome"[Title/Abstract] OR "ARDS"[Title/Abstract]) AND ("Prone Positions"[Title/Abstract] OR "Prone Position"[Title/Abstract] OR "pron*"[Title/Abstract]) AND ("Pressure Ulcer"[Title/Abstract] OR "Pressure Ulcers"[Title/Abstract] OR "Ulcer"[Title/Abstract] OR "Pressure"[Title/Abstract] OR "bedsore*"[Title/Abstract] OR "decubit*"[Title/Abstract])

Cinahl n: 300

#1	AB (“Distress Syndrome, Respiratory” OR “Distress Syndromes, Respiratory” OR “respiratory distress syndromes” OR “Syndrome, Respiratory Distress” OR “Shock Lung” OR “Lung, Shock” OR “Respiratory Distress Syndrome, Acute” OR “acute respiratory distress syndrome” OR “ARDS”)
#2	AB (“Prone Positions” OR “Prone Position” OR Pron*)
#3	AB (“Pressure Ulcer” OR “Pressure Ulcers” OR Ulcer OR Pressure OR Bedsore* OR Decubit*)
#4	AB (“Distress Syndrome, Respiratory” OR “Distress Syndromes, Respiratory” OR “respiratory distress syndromes” OR “Syndrome, Respiratory Distress” OR “Shock Lung” OR “Lung, Shock” OR “Respiratory Distress Syndrome, Acute” OR “acute respiratory distress syndrome” OR

	“ARDS”) AND AB (“Prone Positions” OR “Prone Position” OR Pron*) AND AB (“Pressure Ulcer” OR “Pressure Ulcers” OR Ulcer OR Pressure OR Bedsore* OR Decubit*)

Web of science n: 866

#1	(“Distress Syndrome, Respiratory” OR “Distress Syndromes, Respiratory” OR “respiratory distress syndromes” OR “Syndrome, Respiratory Distress” OR “Shock Lung” OR “Lung, Shock” OR “Respiratory Distress Syndrome, Acute” OR “acute respiratory distress syndrome” OR “ARDS”)
#2	(“Prone Positions” OR “Prone Position” OR Pron*)
#3	(“Pressure Ulcer” OR “Pressure Ulcers” OR Ulcer OR Pressure OR Bedsore* OR Decubit*)
#4	(“Distress Syndrome, Respiratory” OR “Distress Syndromes, Respiratory” OR “respiratory distress syndromes” OR “Syndrome, Respiratory Distress” OR “Shock Lung” OR “Lung, Shock” OR “Respiratory Distress Syndrome, Acute” OR “acute respiratory distress syndrome” OR “ARDS”) AND (“Prone Positions” OR “Prone Position” OR Pron*) AND (“Pressure Ulcer” OR “Pressure Ulcers” OR Ulcer OR Pressure OR Bedsore* OR Decubit*)

Scopus n: 1201

#1	(TITLE-ABS-KEY ("Distress Syndrome, Respiratory" OR "Distress Syndromes, Respiratory" OR "respiratory distress syndromes" OR "Syndrome, Respiratory Distress" OR "Shock Lung" OR "Lung, Shock" OR "Respiratory Distress Syndrome, Acute" OR "acute respiratory distress syndrome" OR "ARDS")
#2	TITLE-ABS-KEY ("Prone Positions" OR "Prone Position" OR pronation)
#4	TITLE-ABS-KEY ("Pressure Ulcer" OR "Pressure Ulcers" OR ulcer OR pressure OR bedsore OR bedsores OR decubitus))
#5	(TITLE-ABS-KEY ("Distress Syndrome, Respiratory" OR "Distress Syndromes, Respiratory" OR "respiratory distress syndromes" OR "Syndrome, Respiratory Distress" OR "Shock Lung" OR "Lung, Shock" OR "Respiratory Distress Syndrome, Acute" OR "acute respiratory distress syndrome" OR "ARDS") AND TITLE-ABS-KEY ("Prone Positions" OR "Prone Position" OR pronation) AND TITLE-ABS-KEY ("Pressure Ulcer" OR "Pressure Ulcers" OR ulcer OR pressure OR bedsore OR bedsores OR decubitus))

Cochrane Library n: 310

#1	("distress syndrome respiratory" OR "distress syndromes respiratory" OR "respiratory distress syndromes" OR "syndrome respiratory distress" OR "shock lung" OR "lung shock" OR "respiratory distress syndrome acute" OR "acute respiratory distress syndrome" OR "ARDS"))
#2	("prone positions" OR "prone position" OR pron*))
#3	("pressure ulcer" OR "pressure ulcers" OR "ulcer" OR "pressure" OR bedsore* OR decubit*))
#4	("distress syndrome respiratory" OR "distress syndromes respiratory" OR "respiratory distress syndromes" OR "syndrome respiratory distress" OR "shock lung" OR "lung shock" OR "respiratory distress syndrome acute" OR "acute respiratory distress syndrome" OR "ARDS") AND ("prone positions" OR "prone position" OR pron*) AND ("pressure ulcer" OR "pressure ulcers" OR "ulcer" OR "pressure" OR bedsore* OR decubit*)

Total articles found	3484
